# Supplementary material for: Drug repurposing for aging research using model organisms
Source: Aging Cell. 2017 Jun 16;16(5):1006–15. doi: 10.1111/acel.12626 (PMC5595691; doi:10.1111/acel.12626)
Supplement: Supplementary file 7 — Data S1 Zip‐Archive of all report cards. [file ACEL-16-1006-s007.zip › RC_TBY.pdf]

TBY

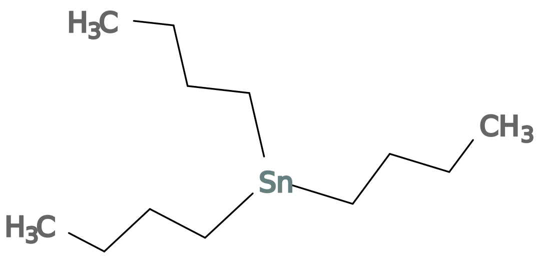

Database identifiers

CHEBI	27086
eMolecules	490549

Ranking

	Rank	Score
Drosophila	371/697	0.402
C. elegans	NA	NA

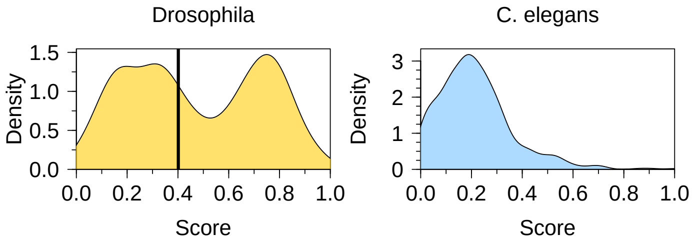

	Ageing implication	Domain conservation	Binding site conservation	Binding affinity	Bioavailability	Lipinski	Promiscuity	Purchasability	Drug approval	**Total**
Drosophila	0.624	0.649	0.854	0.729	(0.9)	0.0	-0.0	0.1	0.075	0.402
C. elegans	NA	NA	NA	NA	NA	NA	NA	NA	NA	NA

Names

- **tributylstannane**
- SnBu3H
- TBT
- Tributyltin

Roles

ChEBI entry 27086 has no roles

Status

Number of Rule of 5 violations	0
Binding affinity to original target in log units (RF-Score prediction)	5.99
Burns *C. elegans* bioavailability prediction	0.46

Compound Target Characteristics

Retinoic acid receptor RXR-alpha

Best gene implication in ageing for this target family came from gene Q0VJ96 via mapping the annotation from RGD 3610 annotated in RGD 2014-03-11. Annotation GO 7568 (aging) was Inferred from Expression Pattern

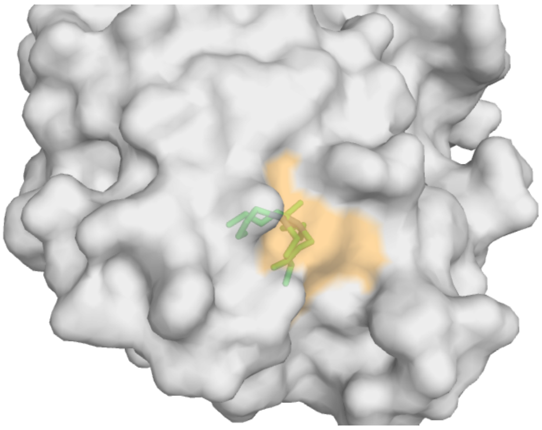

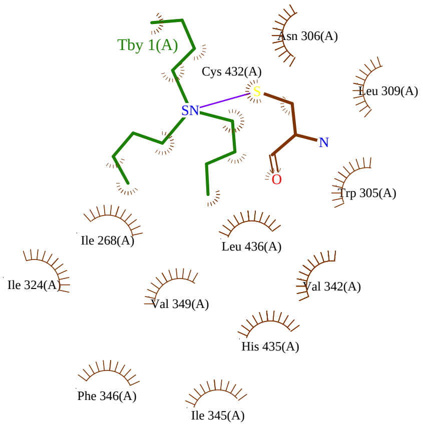

protein	amino acids contacts (binding site)											
PDB:3e94:chainA:P19793	I	W	N	L	I	V	I	F	V	C	H	L
tr:B3KY83:B3KY83_HUMAN	I	W	N	L	I	V	I	F	V	C	H	L
sp:P19793:RXRA_HUMAN	I	W	N	L	I	V	I	F	V	C	H	L
tr:F1D8Q5:F1D8Q5_HUMAN	I	W	N	L	I	V	I	F	V	C	H	L
tr:Q6P3U7:Q6P3U7_HUMAN	I	W	N	L	I	V	I	F	V	C	H	L
tr:Q0VJ96:Q0VJ96_RAT	I	W	N	L	I	V	I	F	V	C	H	L
tr:Q6LC96:Q6LC96_MOUSE	I	W	N	L	I	V	I	F	V	C	H	L
sp:P28700:RXRA_MOUSE	I	W	N	L	I	V	I	F	V	C	H	L
tr:A2AJP1:A2AJP1_MOUSE	I	W	N	L	I	V	I	F	V	C	H	L
sp:P20153:USP_DROME	L	W	I	L	L	V	I	F	I	C	H	L

	whole protein		domain-based		contact-based	
protein	ident	simil	ident	simil	ident	simil
PDB:3e94:chainA:P19793	1.0	1.0	1.0	1.0	1.0	1.0
tr:B3KY83:B3KY83_HUMAN	0.79	0.79	1.0	1.0	1.0	1.0
sp:P19793:RXRA_HUMAN	1.0	1.0	1.0	1.0	1.0	1.0
tr:F1D8Q5:F1D8Q5_HUMAN	1.0	1.0	1.0	1.0	1.0	1.0
tr:Q6P3U7:Q6P3U7_HUMAN	0.9	0.9	1.0	1.0	1.0	1.0
tr:Q0VJ96:Q0VJ96_RAT	0.97	0.99	1.0	1.0	1.0	1.0
tr:Q6LC96:Q6LC96_MOUSE	0.92	0.93	1.0	1.0	1.0	1.0
sp:P28700:RXRA_MOUSE	0.97	0.99	1.0	1.0	1.0	1.0
tr:A2AJP1:A2AJP1_MOUSE	0.97	0.99	1.0	1.0	1.0	1.0
sp:P20153:USP_DROME	0.38	0.65	0.4	0.66	0.67	0.85

usp (FBgn0003964) associated phenotypes

circadian rhythm defective, germline clone, heat sensitive, hypoactive, lethal - all die before end of pupal stage, lethal - all die during P-stage, lethal - all die during first instar larval stage, maternal effect, neuroanatomy defective, partially, rescuable maternal effect, somatic clone, some die during pupal stage, touch sensitivity defective

(Information from FlyBase)

usp (UniProt:P20153) annotation

Function: Receptor for ecdysone. May be an important modulator of insect metamorphosis. Plays an important part in embryonic and post-embryonic development. Binds to ecdysone response elements (ECRES) such as in the promoter region of s15 chorion gene.
Subunit: Heterodimer of USP and ECR. Only the heterodimer is capable of high-affinity binding

to ecdysone. (PubMed:8247157).
Subcellular location: Nucleus.
Domain: Composed of three domains: a modulating N-terminal domain, a DNA-binding domain and a C-terminal ligand-binding domain.

(Information from UniProt)
